# The Impact of Molluscum Contagiosum on Depression, Anxiety and Quality of Life in Children

**DOI:** 10.1111/1346-8138.70443

**Published:** 2026-08-21

**Authors:** Abdulbaki Artik, Hatice Atas, Tugcan Yuksek, Erman Atas, Bahar Oztelcan Gunduz, Huseyin Ilgin Bal, Selda Pelin Kartal, Halenur Teke, Burak Açıkel, Mustafa Sırrı Kotanoğlu, Mehri Durak

**Affiliations:** ^1^ Child and Adolescent Psychiatry Department, Gulhane Training and Research Hospital University of Health Sciences Ankara Turkey; ^2^ Dermatology and Venereology Department Etlik City Hospital Ankara Turkey; ^3^ General Pediatric Department, Gulhane Training and Research Hospital University of Health Sciences Ankara Turkey; ^4^ General Pediatric Department Etlik City Hospital Ankara Turkey; ^5^ Child and Adolescent Psychiatry Department Ankara University Faculty of Medicine Ankara Turkey; ^6^ Ankara Etlik City Hospital Ankara Turkey

**Keywords:** anxiety, child, depression, molluscum contagiosum, quality of life

## Abstract

This study aimed to compare depressive symptoms, anxiety symptoms, and health‐related quality of life between children with molluscum contagiosum and healthy controls and to examine whether lesion number and anatomical localization were associated with these outcomes. This prospectively conducted cross‐sectional study included 72 children with molluscum contagiosum and 80 age‐ and sex‐matched healthy controls aged 6–12 years. Depressive symptoms, anxiety symptoms, and health‐related quality of life were assessed using validated pediatric instruments. Psychological outcomes were also compared according to lesion number and anatomical localization. Children with molluscum contagiosum had significantly higher depression and anxiety scores and significantly lower total quality‐of‐life scores than healthy controls. Depression and anxiety scores differed significantly according to lesion localization. Children with lesions involving highly visible areas, including the face, periocular region, lips, and hands, had higher depression and anxiety scores than those with lesions on the trunk and legs. Lesion number was not significantly associated with depression, anxiety, or quality‐of‐life scores. Female sex was independently associated with approximately twofold higher odds of an elevated anxiety score. Children with molluscum contagiosum may experience greater depressive and anxiety symptoms and poorer health‐related quality of life than healthy peers. Psychological burden appears to be more closely related to lesion visibility and anatomical localization than to lesion number alone. These findings support consideration of psychological screening, particularly in children with highly visible lesions, and highlight the relevance of a broader psychodermatological approach.

## 1 Introduction

Molluscum contagiosum (MC) is a highly contagious viral infection that predominantly affects children, causing lesions on both the skin and mucous membranes. Molluscum lesions typically exhibit a distinct appearance characterized by a central dell and a pearly papule [1]. These lesions commonly appear lighter or skin‐colored and vary in size from 1 to 3 mm. In some cases, giant lesions may develop, extending into the subcutaneous tissue and mimicking cysts or abscesses [2]. The overall prevalence of MC is 8.28%, with higher frequencies observed in warm climates [3]. The global incidence of this infection has been steadily increasing, with a similar trend observed in the United States, where it has risen by up to 20% due to immunocompromised children with HIV and leukemia since the 2000s [4]. The transmission of this virus primarily occurs through close physical contact, contact with contaminated surfaces or objects (fomites), and self‐inoculation, wherein the virus is transferred from one area to another within the infected individual [5]. School‐aged children are particularly susceptible to this infection, highlighting its significance in pediatric populations worldwide [5]. The lesions can appear on various parts of the body, with the number of lesions varying widely among individuals [4].

Children with a higher number of MC lesions are more likely to experience significant impacts on their overall quality of life. Research suggests that approximately 10% of children with MC virus infection encounter substantial impairments in their daily lives [6]. Adults with MC may also experience a decrease in quality of life because of self‐consciousness, disturbing physical symptoms, unsightly appearance, embarrassment of lesions such as anogenital, fear of contamination of sexual partner(s), and negative effects of pain and itching [7].

The connection between dermatological disorders and psychological stress is well‐established, indicating an intricate relationship between psychiatry and dermatology [8]. The visible nature of dermatological conditions frequently results in cosmetic disfigurement, which can substantially impact psychological health, leading to psychopathological issues that affect not only the patient but also their family and society as a whole [9]. This interplay between psychiatry and skin diseases is bidirectional, as psychiatric comorbidity can influence the development and progression of dermatological conditions through the effects of stress, depression, and anxiety [10]. Conversely, dermatological disorders that cause cosmetic disfigurement can significantly impact the psychosocial well‐being of the affected individuals [11].

The relationship between dermatological diseases and psychological health has become an increasingly important focus of psychodermatology, a field that emphasizes the bidirectional interaction between skin disorders and mental well‐being [8, 12, 13]. Psychological stress, anxiety, and depression may influence the onset, severity, and course of dermatological diseases through neuroendocrine and immunological mechanisms, whereas visible skin disorders may adversely affect emotional well‐being, self‐esteem, social functioning, and overall quality of life [10].

Recent psychodermatology research has demonstrated that visible skin diseases affect a wide spectrum of psychological constructs extending beyond depression and anxiety [14, 15]. These include social appearance anxiety, body image disturbance, appearance‐related distress, fear of negative social evaluation, and perceived stigmatization [16]. Social appearance anxiety refers to the distress experienced when individuals fear being negatively judged because of their physical appearance [17]. In a cross‐sectional controlled study of adults with facial acne, patients reported greater social appearance anxiety than healthy controls, illustrating how lesions on highly visible areas may affect concerns about appearance and social evaluation [18]. Body image disturbance refers to negative perceptions, thoughts, or feelings about one's physical appearance, whereas appearance‐related distress reflects the broader emotional discomfort arising from concerns about visible physical differences [19]. Perceived stigmatization refers to the individual's sense of being judged, avoided, rejected, or treated differently because of a visible skin condition [20].

Among the factors contributing to psychological distress, lesion visibility appears to play a particularly important role [21]. Lesions located on exposed body regions, such as the face, lips, periocular area, and hands, are more likely to attract attention during daily social interactions [22]. Visible skin lesions may increase self‐consciousness, embarrassment, avoidance of social activities, and fear of negative evaluation [15, 17].

For children, the social consequences of visible lesions may be especially important. Staring, teasing, bullying, peer rejection, and avoidance by others may reinforce perceived stigmatization, particularly when the condition is regarded as contagious or unhygienic. These experiences may contribute to shame, social exclusion, reduced self‐esteem, and withdrawal from school or recreational activities. The psychosocial burden of visible skin disease may therefore depend not only on objective disease severity but also on how noticeable and stigmatizing the child perceives the lesions to be [23, 24].

The close relationship between dermatological disorders and psychiatric symptoms has been well documented in conditions such as psoriasis, vitiligo, alopecia areata, and acne vulgaris. Numerous studies have reported higher levels of depression and anxiety symptoms and poorer quality of life in these disorders. However, despite the high prevalence of MC in childhood, relatively little attention has been paid to its psychological consequences. Existing studies have mainly focused on health‐related quality of life [25], while evidence regarding depression and anxiety in pediatric MC populations remains limited. Moreover, the potential contribution of lesion localization and visibility to psychological outcomes has not been adequately explored.

Depression and anxiety were selected as the primary psychological outcomes because they represent clinically important dimensions of internalizing distress and are directly relevant to psychiatric assessment and intervention in children. In addition, previous studies of pediatric MC have focused predominantly on health‐related quality of life, whereas depressive and anxiety symptoms have received comparatively limited attention. Validated pediatric instruments were also available to assess both constructs in the target age group. Nevertheless, appearance‐related constructs, including social appearance anxiety, body image disturbance, appearance‐related distress, perceived stigmatization, and fear of negative evaluation, may represent more specific psychosocial processes through which visible lesions affect emotional well‐being and social functioning. Because these constructs were not directly assessed in the present study, they were considered in the interpretation of our findings and are highlighted as important areas for future research. Therefore, the present study aimed to compare depression, anxiety, and quality of life between children with MC and healthy controls and to examine whether lesion number and anatomical localization were associated with these outcomes.

## 2 Methods

### 2.1 Study Population and Study Design

The study was conducted prospectively between September 15, 2022, and December 15, 2022, at the Dermatology outpatient clinic of Gülhane Training and Research Hospital. Participants included children aged 6 to 12 years diagnosed with MC, along with healthy controls matched by age and gender. Inclusion criteria for the study required patients to have experienced symptoms of MC for at least 3 months, while exclusion criteria involved significant physical or neurological illnesses (e.g., heart problems, epilepsy), pervasive developmental disorders, intellectual disability, or a diagnosis of psychotic disorders. Patients receiving treatment for systemic diseases were also excluded. We also assessed whether the children in both the patient and control groups were taking psychiatric medication. Patients using psychiatric medications were excluded from the study. A total of 72 outpatient cases with MC and 80 healthy children were included in the study. Before enrollment, all participants and their parents received a detailed patient information form and provided written informed consent. Following a dermatological examination, patients were referred to a child and adolescent psychiatrist.

### 2.2 Tools

Depression, anxiety, and quality of life scales given to the patients were filled out and evaluated by a child and adolescent psychiatrist.

### 2.3 Childhood Depression Inventory (CDI)

CDI is a self‐assessment scale applicable to children between the ages of 6 and 17. For this study, the scale has been filled in by the child him/herself. There were three different options for each item on the 27‐point scale. The child is asked to choose the most appropriate sentence for the last 2 weeks. Each item takes 0, 1, or 2 points according to the severity of the indication. The maximum score is 54, with higher scores indicating more severe depression. The cutoff score is recommended as 19. The validity and reliability of the inventory in Turkish have also been established [26].

### 2.4 State–Trait Anxiety Inventory for Children

This anxiety scale that is developed by Spielberger for children, consists of two parts with 20 questions. Each item is scored on a scale of 1 to 3, based on the presence and severity of the symptom, with total scores ranging from 20 to 60. The validity and reliability of the inventory in Turkish have also been established [27].

### 2.5 PedsQL 4.0 Generic Core Scales

The PedsQL 4.0 Generic Core Scales were designed by [28] in 2001 as an HRQOL measurement for children and adolescents aged between 2 and 18 years. It consists of 23 items: Physical functioning (8 items), emotional functioning (5 items), social functioning (5 items), and school functioning (5 items). Child self‐reports include 3 age groups: 5–7 years (young children), 8–12 years (children), and 13–18 years (teens). The parent proxy report, however, includes 4 age groups: 2–4 (toddlers), 5–7, 8–12, and 13–18. The response scale is a 5‐point Likert scale for all age groups, except for the 5‐ to 7‐year‐old version. Items are reverse‐scored and linearly transformed to a 0–100 scale, where higher scores indicate better HRQOL. The response scale of the 5‐ to 7‐year‐old version is completed with the help of an interviewer and simplified to a 3‐point scale (0, 2, and 4 points). The child answers the items with the help of a visual scale (happy, neutral, and sad faces). The validity and reliability of the inventory in Turkish have also been established [29]. Both physical and psychosocial functionality are assessed by the parent and child versions of the scale. In this study, the PedsQL‐P questionnaire was primarily completed by mothers or other caregivers if mothers were unavailable.

### 2.6 Data Analysis

The data were analyzed using the IBM SPSS Statistics Standard Concurrent User V26 (IBM Corp., Armonk, New York, USA) statistical package program. Descriptive statistics were presented as mean ± standard deviation, median, and interquartile range values. The normal distribution of data pertaining to scale scores was assessed using the Shapiro–Wilk normality test. For the comparison of scale scores between the patient and control groups, an independent samples *t*‐test was used for normally distributed data, and the Mann–Whitney U test was used for non‐normally distributed data. Scale scores based on the number and location of lesions were compared using one‐way analysis of variance (ANOVA) or Kruskal–Wallis analysis, depending on the normality of the data. For post hoc multiple comparisons, Tukey's Honestly Significant Difference (HSD) test or Tamhane test were used for one‐way ANOVA. Variables identified in the univariate analyses were entered into a logistic regression model to determine independent predictors of an anxiety score above 29. A *p*‐value of less than 0.05 was considered statistically significant.

## 3 Results

The patient group exhibited significantly lower scores in total quality of life (*p* < 0.001) and significantly higher scores in both depression (*p* < 0.001) and anxiety (*p* < 0.001) compared to the control group (Table 1). Statistically significant differences were observed in depression (*p* < 0.001) and anxiety scores (p < 0.001) based on lesion locations; however, no significant difference was found in total quality of life scores (*p* = 0.70). Patients with lesions involving the face, periocular region, lips, and hands had significantly higher anxiety and depression scores than those with lesions on the trunk and legs (Table 2) (See Figures 1 and 2). While lower QOL/higher anxiety‐depression scores were observed with an increasing number of lesions, these differences did not reach statistical significance (Table 3). Female sex was independently associated with approximately twofold higher odds of an anxiety score above 29 (*p* = 0.04, OR = 2.00, 95% CI: 1.1–3.9) (Table 4).

**TABLE 1:** Comparison of scale scores between Patient and Control Groups.

	Groups				*p*
	Control		Patient		
	Mean (SD)	Median	Mean (SD)	Median	
Total Quality of Life Score	43.9 (8.7)	44.0	30.6 (7.3)	30.5	**< 0.001** ^a^
Anxiety Score	28.2 (5.3)	28.0	37.7 (9.0)	38.0	**< 0.001** ^b^
Depression Scores	22.9 (3.8)	24.0	38.7 (8.9)	37.0	**< 0.001** ^**b**^

*Note:* Data are given as median (interquartile range) or mean ± standard deviation. Bold values indicate statistically significant differences between the patient and control groups (*p* < 0.05).

^a^ Independent samples *t*‐test.

^b^ Mann–Whitney U test.

**TABLE 2:** Comparison of scale scores by lesion locations.

	*n*	Total Quality of Life Score		*p* ^a^	Depression Score		*p* ^c^	*p* ^b^	Anxiety Score		*p* ^d^	*p* ^b^
		Mean (SD)	Median		Mean (SD)	Median			Mean (SD)	Median		
Head, Face	10	28.2 (5.9)	27.5	0.70	50.2 (1.8)	50.0	—	< 0.001	46.6 (5.6)	45.0		< 0.001
Lips, Eyes	8	28.8 (5.7)	29.0		49.9 (2.2)	50.0	1.00		46.4 (7.3)	44.5		
Neck	10	31.5 (10.4)	30.5		32.6 (7.5)	32.0	0.001		30.4 (4.4)	28.5		
Trunk	19	30.5 (8.3)	30		34.6 (5.3)	34.0	< 0.001		34.56 (7.5)	36.0		
Arm	14	33.0 (5.3)	34		33.6 (4.6)	34.5	< 0.001		34.0 (5.3)	35.5		
Hand	5	30.8 (6.3)	32		47.2 (6.6)	52.0	1.00		49.4 (6.4)	50.0		
Legs	6	29.8 (7.2)	33		32.7 (5.6)	34.0	0.01		32.8 (6.2)	32.0		
Control	80	44.0 (8.7)			22.9 (3.8)				28.2 (5.3)	28.0		

*Note:* Data are given as median (interquartile range) or mean ± standard deviation.

^a^ Kruskal–Wallis test.

^b^ One‐way analysis of variance.

^c^ Tamhane test (levene *p* = 0.002).

^d^ Tukey test (Levene *p* = 0.812).

**FIGURE 1:**
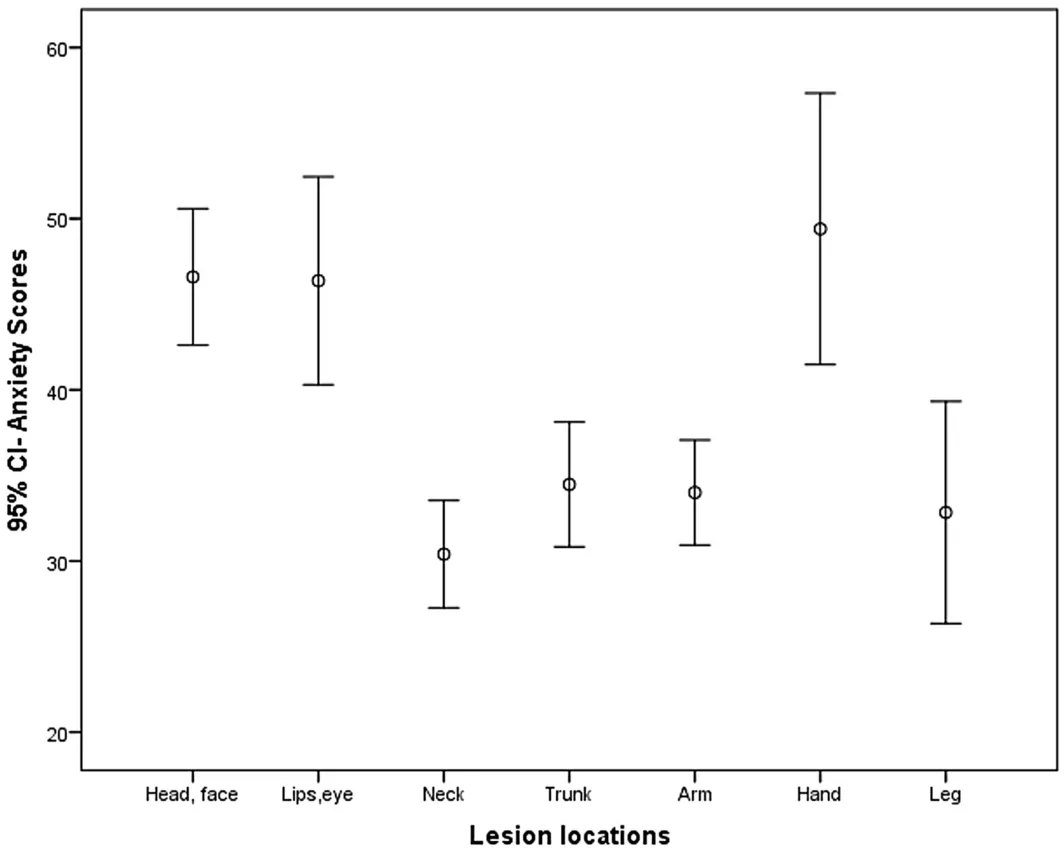
Error bar of anxiety scores according to lesion localizations.

**FIGURE 2:**
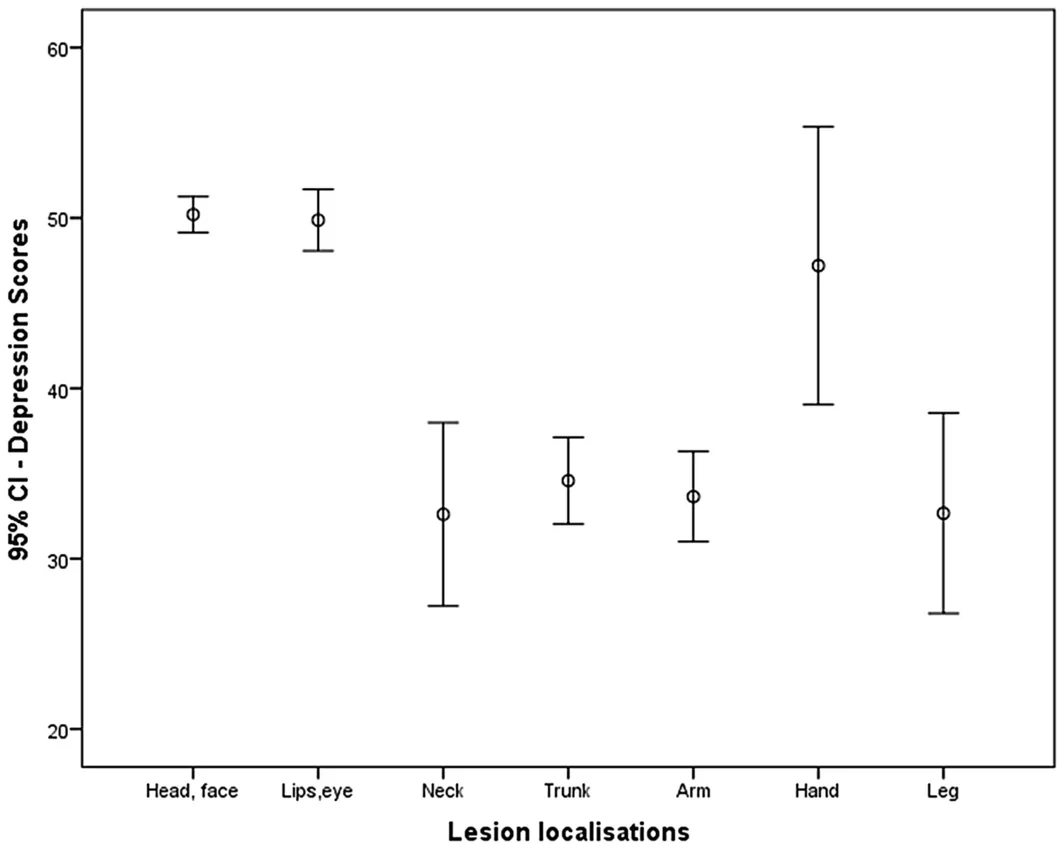
Error bar of depression scores according to lesion localizations.

**TABLE 3:** Comparison of scale scores by number of lesions.

Number of lesions	*n*	Total Quality of Life Score		*p* ^a^	Depression scores		*p* ^b^	Anxiety scores		*p*
		Mean (SD)	Median		Mean (SD)	Median		Mean (SD)	Median	
1–5	26	30.0 (6.4)	32	0.92	38.7 (9.0)	37.5	0.10	37.4 (8.1)	36.0	0.46
5–10	29	30.6 (7.9)	30		40.9 (8.6)	38		39.2 (10.6)	39.0	
10–50	17	31.6 (7.7)	30		35.0 (8.5)	34		35.8 (7.1)	38	
Control	80	43.9 (8.7)	44.0		22.9 (3.8)	24.0		28.2 (5.3)	28.0	

*Note:* Data are given as median (interquartile range) or mean ± standard deviation.

^a^ Kruskal–Wallis test.

^b^ One‐way analysis of variance.

**TABLE 4:** Logistic regression analysis of predictors of increased anxiety.

Parameters	Unit	*p*	OR	95% CI
Gender	Female/Male	0.04	2	1.1–3.9
Family history	Yes/No	0.75	0.75	0.1–4.3
Localization		0.14	0.75	0.5–1.1
Localization	Head, Face, lips, eyes, hands/others	0.99	0.1	0.1–1.1
Number	1–4/5–10/11–50	0.68	0.86	0.4–1.8
Age		0.12	0.82	0.6–1.1

## 4 Discussion

The present study compared depression and anxiety symptoms and health‐related quality of life between children with molluscum contagiosum and healthy controls. Children with MC had significantly higher depression and anxiety scores and poorer quality of life than healthy children. Lesion localization was also associated with psychological outcomes: Children with lesions involving highly visible areas, including the face, lips, periocular region, and hands, had higher depression and anxiety scores than those with lesions located on less visible body regions. In contrast, lesion count was not significantly associated with depression, anxiety, or quality‐of‐life scores. Taken together, these findings suggest that the psychological burden of MC may be related not only to the presence or extent of the disease, but also to the visibility and anatomical location of the lesions.

Previous studies in children with MC have primarily focused on health‐related quality of life, while evidence regarding depression and anxiety remains limited. To our knowledge, the present study is among the first prospective cross‐sectional investigations to evaluate depression, anxiety, and quality of life simultaneously in children with MC while also examining the associations of lesion number and localization with psychological outcomes. These findings extend the existing MC literature by indicating that lesion visibility may be clinically more relevant to emotional distress than lesion count alone. These findings also support the need to interpret pediatric MC within a broader psychodermatological framework rather than considering it solely as a benign and self‐limiting cutaneous infection.

In this context, “visibility burden” refers to the disproportionate psychosocial impact of lesions located on highly visible body areas, which may exceed the burden suggested by lesion number or overall disease extent alone. Previous studies have similarly shown that children and adolescents with visible dermatological conditions may experience substantial psychological and quality‐of‐life burden. For example, a controlled study of adults with contact dermatitis reported greater social appearance anxiety than in healthy controls, supporting the view that visible inflammatory skin lesions may heighten concerns about appearance and negative social evaluation [17]. Comparable findings have been reported in a case–control study of adults with seborrheic dermatitis, in which social appearance anxiety was elevated [16]. A comparative study reported higher social appearance anxiety in adults with verruca vulgaris than in healthy controls [15]. Although the study involved an adult population, its findings are relevant to the present results because both conditions involve visible viral skin lesions with similar appearance‐related and social implications. A comparative cross‐sectional study of adults with alopecia areata likewise highlighted the psychological burden associated with visible changes in appearance, particularly social appearance anxiety [14]. Although alopecia areata differs clinically from MC, the study supports the broader relevance of lesion visibility and appearance‐related social evaluation to psychological distress. Pediatric vitiligo has been associated with higher levels of depression and anxiety and poorer health‐related quality of life compared with healthy peers [30, 31], while systematic reviews have demonstrated impaired quality of life in children and adolescents with alopecia areata and psoriasis [32, 33]. Population‐based studies have also reported associations of alopecia areata and psoriasis with major depressive disorder [34, 35]. Beyond these clinical outcomes, visible skin diseases have been associated with body image disturbance, reduced self‐esteem, perceived stigmatization, social appearance anxiety, emotional distress, social withdrawal, and poorer quality of life [19, 20, 36]. These appearance‐related psychosocial processes may contribute to increased vulnerability to depressive and anxiety symptoms.

In our study, children with a higher number of MC lesions did not appear to be at an increased risk of experiencing significant adverse effects on their quality of life. However, previous research has reported that approximately 10% of children with MC experience severe impairments in quality of life [25]. Factors influencing QOL include exclusion from school or extracurricular activities, lesion visibility, lesion count, parental concern, and the presence of pain, tenderness, and itching [37]. These findings suggest that quality‐of‐life impairment may not be determined by lesion count alone. In particular, lesions located on highly visible body areas may have a disproportionate impact by increasing embarrassment, social avoidance, fear of negative evaluation, and perceived stigmatization. By contrast, lesions located on less visible or usually covered body areas may be less likely to attract unwanted social attention or directly affect appearance‐related concerns, although they may still contribute to disease‐related discomfort and quality‐of‐life impairment. These psychosocial experiences may also contribute to broader emotional distress, including depressive and anxiety symptoms. Quality‐of‐life impairment may arise through several interrelated psychosocial pathways. Appearance‐related self‐consciousness, fear of negative evaluation, perceived stigmatization, and concerns about contagion may lead children to avoid peer interactions, school activities, sports, or recreational settings. Reduced participation and difficulties in social relationships may, in turn, impair emotional, social, and school functioning, which are central components of pediatric health‐related quality of life.

Visible lesions may produce greater psychological burden because they are difficult to conceal and remain noticeable during face‐to‐face interactions. Lesions involving the face, periocular area, lips, and hands may attract unwanted attention, heighten self‐consciousness, and increase concern about how the child is perceived by others. Hand lesions may be particularly noticeable during play, writing, eating, school activities, and physical contact with peers. By contrast, lesions on covered body areas are more easily concealed and may therefore attract less repeated social attention during everyday interactions. Consistent with our findings, a case–control study of facial vitiligo reported elevated social appearance anxiety in patients with facial involvement. The study also found that periorbital and perioral lesions were particularly distressing, supporting the clinical relevance of lesion localization within highly visible facial areas [22]. Similar findings have been reported in a case–control study of adults with pemphigus vulgaris, in which lesion visibility and localization were associated with social appearance anxiety [21]. Although pemphigus vulgaris differs clinically from MC, these findings further support the importance of anatomical lesion location in appearance‐related psychological burden. This may be especially relevant during childhood and early adolescence, when physical appearance, peer acceptance, and social belonging become increasingly important. Visible lesions may also alter the child's self‐image by increasing dissatisfaction with appearance and the sense of being different from peers. A more negative self‐image may, in turn, reduce self‐esteem and contribute to social withdrawal, sadness, and greater vulnerability to depressive symptoms [38, 39]. Body image encompasses an individual's perceptions, thoughts, and feelings about their physical appearance. Lesions on highly visible areas may become disproportionately salient within the child's body image, leading the child to focus more strongly on the affected area and to evaluate their overall appearance more negatively. Such body image disturbance may contribute to reduced self‐confidence, heightened sensitivity to social evaluation, and emotional distress [19]. In school and peer settings, these experiences may contribute to body image dissatisfaction, social appearance anxiety, embarrassment, fear of negative evaluation, and avoidance of social activities. Children may attempt to reduce anticipated scrutiny by concealing affected areas, avoiding eye contact, limiting participation in school or recreational activities, or withdrawing from peer interactions. Although these behaviors may temporarily reduce discomfort, they may also restrict social participation and reinforce appearance‐related anxiety over time. Concerns about contagion may further intensify these reactions, particularly when peers or adults misunderstand the condition. Over time, these appearance‐related experiences may impair self‐esteem and social functioning and may represent possible psychosocial pathways linking visible lesions with depressive and anxiety symptoms and poorer quality of life [19, 20, 25, 36]. However, because self‐image, body image, and the other appearance‐related constructs were not directly assessed in the present study, their possible mediating roles could not be tested and should be regarded as plausible psychosocial explanations rather than established mechanisms.

Stigmatization represents another important component of the psychological burden associated with visible skin disease. Children with conspicuous lesions may experience staring, unwanted questions, teasing, avoidance, or peer rejection, particularly when the condition is incorrectly perceived as unhygienic or highly contagious. Such reactions may generate embarrassment, shame, and a sense of social exclusion, thereby reducing participation in school, recreational activities, and peer relationships. School‐related embarrassment and peer rejection may therefore represent important psychosocial pathways linking visible lesions with emotional distress and impaired quality of life. Visible lesions may also alter everyday peer interactions by making children more reluctant to initiate contact, participate in group activities, or engage in situations involving close physical proximity. Repeated staring, questions, teasing, or avoidance by peers may weaken the child's sense of belonging and make it more difficult to establish or maintain supportive friendships. Over time, these experiences may impair social confidence and broader social functioning. Perceived stigmatization may also reinforce appearance‐related avoidance and fear of negative social evaluation, contributing to emotional distress and impaired quality of life. Importantly, the psychological burden of visible dermatological conditions may depend not only on objective disease severity but also on how noticeable and stigmatizing patients perceive their lesions to be [20, 38, 40, 41, 42].

In the present study, female sex was independently associated with approximately twofold higher odds of elevated anxiety scores. Sex‐ and gender‐related differences in the psychosocial burden of dermatological conditions have been reported, although findings are not entirely consistent [43, 44]. Studies in adults with skin disease have suggested that women may experience greater body dysmorphic concerns and appearance‐related psychological burden [45]. Sex‐related differences have also been reported in stigmatization, coping, and social appearance anxiety [44]. These findings raise the possibility that body image concerns, sensitivity to appearance‐related social evaluation, and perceived stigmatization may partly contribute to the higher anxiety scores observed among girls with visible MC lesions [44, 45, 46]. However, these mechanisms were not directly assessed in the present study, and the available supporting evidence is derived predominantly from adult dermatology populations. Therefore, this finding should be interpreted cautiously and examined further in pediatric studies.

Taken together, these findings suggest that the psychological burden of MC is not limited to its physical manifestations. Children with MC had higher depression and anxiety scores and poorer quality of life than healthy controls, while lesion location appeared to be more closely related to emotional outcomes than lesion count. Lesions on highly visible areas may draw greater attention during social interactions and increase appearance‐related concerns. Taken together, these findings suggest that appearance‐related psychological burden may be an important component of the emotional impact of MC, particularly when lesions involve highly visible areas. Lesions on less visible areas may carry a lower appearance‐related and interpersonal burden, which may help explain the lower depression and anxiety scores observed in these localization groups. Concerns about contagion, whether accurate or based on misconception, may also contribute to embarrassment, avoidance by others, perceived stigma, and difficulties in peer relationships. Although these processes were not directly assessed in the present study, they may help explain the greater emotional burden observed in children with visible lesions. Clinical evaluation of children with MC should therefore take into account both disease characteristics and the child's psychosocial experience. A multidisciplinary approach may be particularly useful for children with highly visible lesions or signs of emotional or social difficulty. Dermatologists may identify children at increased psychosocial risk during routine clinical assessment, while child psychiatrists and psychologists may provide further evaluation and support for anxiety, depressive symptoms, appearance‐related distress, social withdrawal, or school‐related problems. Collaboration between these disciplines may facilitate early recognition of difficulties and more appropriate referral when needed.

Several limitations should be considered. The relatively small sample size and single‐center design may limit the generalizability of the findings. In addition, the cross‐sectional design does not allow causal inferences or clarification of the temporal relationship between MC and psychological outcomes. Psychiatric diagnoses were not established using a structured or semi‐structured diagnostic interview; therefore, depression and anxiety scores should be interpreted as measures of symptom severity rather than as diagnostic outcomes. Appearance‐related constructs, including social appearance anxiety, body image disturbance, perceived stigma, fear of negative evaluation, and appearance‐related avoidance, were also not directly assessed. Accordingly, the psychological effects of MC may not be limited to depressive and anxiety symptoms but may also involve self‐image and body image concerns, appearance‐related distress, perceived stigmatization, social withdrawal, and difficulties in peer functioning. As a result, the psychosocial pathways proposed to explain the association between visible lesions and emotional distress remain hypothetical and could not be tested as mediating mechanisms. The study also did not assess patients' subjective perception of lesion visibility, and lesion duration was not examined as a possible factor associated with psychological adaptation. Longer disease duration might increase cumulative appearance‐related and social burden, although some children may develop psychological adaptation over time. Larger, multicenter longitudinal studies using structured diagnostic assessments and validated measures of appearance‐related distress, stigma, body image, and social functioning are needed to clarify these mechanisms and the longer‐term psychological effects of MC.

## 5 Conclusion

Children with molluscum contagiosum had higher depression and anxiety scores and poorer quality of life than healthy controls. The psychological burden was greater when lesions involved highly visible areas, including the face, periocular region, lips, and hands, whereas lesion count appeared to be less informative. Female sex was also associated with higher anxiety scores. These findings indicate that the impact of MC extends beyond its physical manifestations and may include appearance‐related concerns, perceived stigma, and difficulties in social interactions. Routine psychological screening for emotional distress and quality‐of‐life impairment should be considered in children with lesions involving highly visible areas, particularly when social withdrawal or school‐related difficulties are present. A multidisciplinary approach involving dermatology and child mental health services may help identify vulnerable patients and provide appropriate support.

## Funding

The authors have nothing to report.

## Ethics Statement

All procedures were conducted in accordance with the Declaration of Helsinki. The study protocol was approved by the Ethics Committee of Gulhane Training and Research Hospital (reference no. 2022–273).

## Consent

Written informed consent was obtained from the parents or legal guardians of all participants before enrollment.

## Conflicts of Interest

The authors declare no conflicts of interest.

## Data Availability

The data that support the findings of this study are available from the corresponding author upon reasonable request.
